# Flexible PVDF sensors for bruxism bite force measurement: A redefined instrumental approach

**DOI:** 10.1371/journal.pone.0330422

**Published:** 2025-08-21

**Authors:** Bernardo Flores-Ramírez, Ernesto Suaste-Gómez, Víctor García-Limón, Fernando Angeles-Medina

**Affiliations:** 1 Department of Electrical Engineering, Section Bioelectronics, Center for Research and Advanced Studies (CINVESTAV), Mexico City, Mexico; 2 Division of Postgraduate Studies and Research, Odontology Faculty, National Autonomous University of Mexico (UNAM), Mexico City, Mexico; Showa University: Showa Daigaku, JAPAN

## Abstract

Bruxism, characterized by involuntary clenching or grinding teeth, is a prevalent condition primarily associated with oral health consequences such as dental wear, temporomandibular disorders (TMD), and masticatory muscle pain, while also being linked secondarily to tension-type headaches and sleep disturbances. Accurate measurement of bite force is valuable for assessing bruxism severity, understanding its biomechanical effects, and evaluating treatment outcomes. However, existing measurement methods often lack practicality for continuous or real-time monitoring. This study introduces a novel, noninvasive approach using flexible polyvinylidene fluoride (PVDF) capacitive sensors, leveraging PVDF’s piezoelectric properties to correlate bite force with the output frequency of a precision timer circuit. Experimental results demonstrated the sensor’s ability to measure bite forces in two bruxism scenarios. Static tests simulating clenching forces (0−80 kg) demonstrated sensor sensitivities of 0.0051–0.00533 × 10^3^ Hz/kg, while dynamic tests simulating grinding motions (0−24 kg at 1 Hz) showed sensitivities of 0.0052–0.01019 × 10^3^ Hz/kg. While the sensor demonstrated high sensitivity in static loading, its response to rapid force fluctuations was non-linear, suggesting a need for further optimization in dynamic applications. The sensors effectively tracked. real-time force variations while accounting for PVDF’s viscoelastic properties and minimizing thermal effects. The proposed sensor system offers flexibility and biocompatibility, making it ideal for real-time monitoring. This innovative system for measuring bite force in bruxism-simulated scenarios represents a step forward in objectively assessing this masticatory behavior. Future work will focus on integrating the sensor with digital health tools and exploring its use in detecting other valuable variables in the oral cavity.

## Introduction

Polyvinylidene fluoride (PVDF) is a versatile polymer with exceptional piezoelectric, mechanical, and biocompatible properties, making it an ideal material for sensing applications in the dental field [1,2]. Among its five crystalline phases (α, β, γ, δ, and ε), the β-phase is particularly significant due to its high dipole moment, which enhances PVDF’s electromechanical properties. The alignment of dipole moments in the β-phase can be optimized through mechanical stretching or electrical poling, maximizing the material’s piezoelectric, pyroelectric, and ferroelectric capabilities [3,4]. These properties, combined with its flexibility, biocompatibility, and cost-effectiveness, position PVDF as a suitable choice for developing innovative sensors [5–24], particularly for applications like occlusal force measurement for bruxism assessment.

## PVDF as a membrane sensor

PVDF’s unique properties make it highly suitable as a force sensor in dental applications. Its flexibility allows it to conform to the complex geometries of dental structures, enabling seamless integration into wearable devices, dental appliances, or thin films placed directly on teeth [5]. Unlike rigid piezoelectric materials like lead zirconate titanate (PZT) or barium titanate (BaTiO3), PVDF’s mechanical resilience ensures that it can withstand the repetitive loading and unloading cycles associated with occlusal forces without fracturing or losing functionality [6,7].

PVDF membranes exhibit a broad frequency response, making them capable of accurately capturing dynamic forces, such as those generated during chewing or bruxism episodes, typically occurring at a physiological frequency of 1–2 Hz [8–10]. This frequency range is critical for ensuring the sensor’s performance aligns with real-world dental conditions. Additionally, PVDF’s viscoelastic behavior allows it to recover quickly after deformation, ensuring consistent and reliable measurements over time [11].

In addition to its mechanical force sensing capabilities, PVDF’s pyroelectric and ferroelectric properties allow it to detect various physical variables. Its sensitivity to fluctuations in humidity [12,13,14] can be utilized to monitor oral moisture levels, which are essential for assessing conditions such as dry mouth (xerostomia) and evaluating dental prosthetics’ performance. Moreover, the pyroelectric characteristics of PVDF enable it to measure temperature variations, facilitating applications like tracking oral temperature changes during dental procedures or diagnosing inflammatory conditions within the oral cavity [15,16,17,6].

Existing instrumental techniques employed for the assessment of bruxism that involve the force generated by the muscles of the mandible, such as electromyography (EMG) and conventional bite force measurement (BFM) [8,17–24], face notable limitations. While EMG proves effective in measuring muscle activity, it is often invasive, bulky, and uncomfortable for patients, restricting its suitability for prolonged monitoring [8,17,18,25–31]. Similarly, BFM relies on piezoelectric or piezoresistive materials, which are typically rigid films that struggle to conform to the intricate contours of dental cusps. These sensors measure changes in electrical resistance under applied force [32–38]. While effective, these devices are often less sensitive to small force variations or wide-range measurements due to their nonlinear response [32–38].

In contrast, PVDF membranes demonstrate unique adaptability, which could enable them to precisely conform to the complex topography of dental cusps and even envelop entire teeth. This conformability represents a significant advantage and enhanced versatility over traditional piezoresistive sensors employed in vertical force measurement for potentially capturing lateral forces, tongue pressure over the teeth, and subtle dental displacements [39].

The distinctive characteristics of PVDF membranes open new avenues for comprehensive oral health monitoring by integrating dental appliances or wearable devices. Compared to traditional force sensors, they facilitate capturing a wider range of biomechanical data, enabling real-time monitoring and easy integration with digital diagnostic systems, further enhancing their potential clinical utility [7,40].

## Bruxism and bite force

Bruxism, a condition involving repetitive clenching and grinding teeth, is a common disorder that can lead to serious dental and jaw problems, such as tooth deterioration, jaw pain, and temporomandibular joint (TMJ) disorders. Despite its prevalence, bruxism is often underdiagnosed due to the lack of reliable tools to measure its severity [20,21,41–44].

Quantifying bite force—the force exerted during clenching or grinding—is crucial for better understanding and managing this condition. Tracking changes in bite force actively helps clinicians assess the severity of bruxism [45,46], identify patients at risk of dental damage or jaw disorders, and monitor the effectiveness of treatments, such as mouthguards or behavioral therapy.

Additionally, understanding the forces involved in bruxism can shed light on how it damages teeth and jaw structures, guiding the development of better treatments and materials.

According to the literature, the maximum voluntary bite force ranges from 500 N to 750 N in healthy individuals and from 360 N to 1116 N in individuals with temporomandibular disorders or bruxism, depending on factors such as sex, age, health status, pain, number of teeth, occlusion, craniofacial morphology, and the measurement technique employed [33,34,36,39,46–48].

Except for computer simulation techniques, the remaining methods for measuring bite force introduce measurement devices that alter the vertical dimension. These devices modify the natural functioning of the jaw, the actuating muscles, and the periodontal perception. Consequently, the maximum bite force values range depending on the instrument used [45,46,49].

Beyond its clinical applications, measuring bite force can help researchers explore the links between bruxism and other health issues, such as sleep apnea, anxiety, and chronic pain. Early detection and management of bruxism through bite force measurement can prevent long-term damage and reduce the need for costly dental procedures [14,17,35,50,51].

This study focuses on developing and testing a PVDF-based sensor system to measure simulated bite forces characterized by bruxism events. By providing accurate, real-time data, this technology can potentially improve the diagnosis, treatment, and prevention of bruxism, benefit patients and advancing research in dental and craniofacial health.

## 1 Experimental methodology

The proposed solution utilizes the piezoelectric characteristics of poly-vinylidene fluoride (PVDF) membranes to develop a sensor capable of quantifying human bite force during the repetitive and oscillatory patterns characteristic of bruxism activity [26,52,53] by correlating the effect of the membrane capacitance on the frequency response of a precision timer circuit when subjected to known vertical forces.

This approach identifies distinct frequency signatures associated with bruxism, in contrast to the controlled and consistent forces characteristic of normal occlusion.

We used a hydrophobic PVDF microporous membrane (Immbilon-PSQ) to construct two prototype force sensors with copper electrodes. These sensors comprise a pair of copper sheets that adhere to the membrane’s contact surface on both sides.

**Fig 1:**
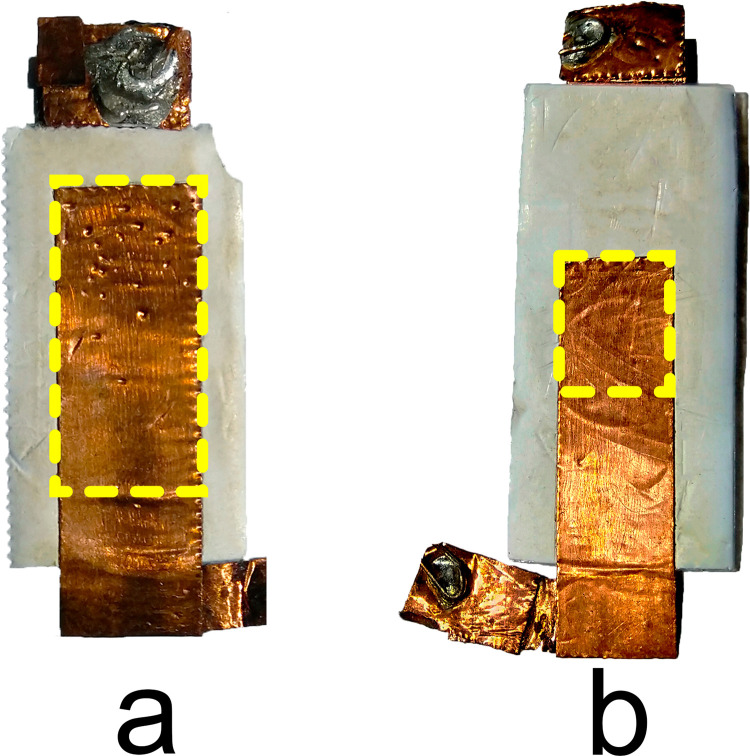
Membrane sensor proposal. The dashed line indicates the active area for **a)** Sensor A and **b)** Sensor **B.**

The sensor dimensions were based on the molar spacing in the Columbia M-860 dental model to ensure anatomical compatibility. Two sensor configurations were designed with 70% and 30% longitudinal electrode overlaps to evaluate the effect of active area size on sensitivity and signal resolution. Table 1 presents the differences between the designs. A larger active area (Sensor A) was expected to provide higher sensitivity, while a smaller active area (Sensor B) was anticipated to offer better spatial resolution. Fig 1 depicts both designs.

**Table 1:** Sensor physical attributes.

Sensor^1^	Longitudinal electrode overlap^2^	Active area^1^ (mm)
A	70%	140
B	30%	60

^1^Membrane size: 30 x 10 (mm).

^2^Effective intersections between electrodes.

PVDF exhibits piezoelectric properties only when its dipoles are aligned under a strong electric field. A voltage multiplier circuit and flyback transformer were used to generate a high-voltage electric field (10 kV) for polarization. This process ensured consistent dipole alignment, enhancing the piezoelectric response of the membranes.

**Fig 2:**
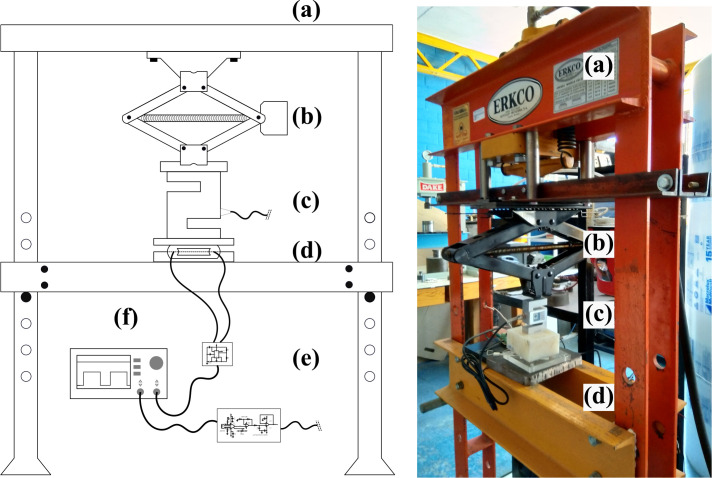
Experimental setup to emulate bruxism behavior. Picture and schematic view of PVDF membranes mounted on a flat surface of a dental arch model coupled to a static or oscillatory base (d) before applying sustained force on a load cell (c) through a spindle mechanism (b) fixed to a steal mainframe **(a)**. Oscilloscope (f) displays the square pulsed signal from each precision timer circuit and the load cell conditioning-circuit output DC voltage **(e)**.

To differentiate the sensor responses to applied sustained vertical force and force exerted during oscillatory motion, we implemented two tests: a static test and a dynamic test, which relate to clenching and grinding behavior. Fig 2 presents the experimental setup for both tests.

**Fig 3:**
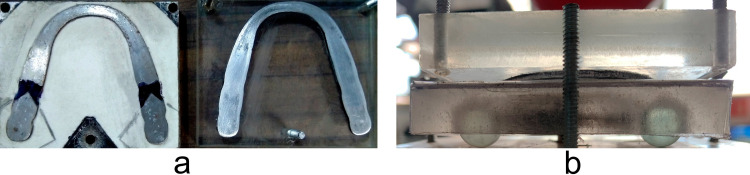
Compression test blocks for static and dynamic tests. **(a)** Steel bottom plate and acrylic top plate for static test. **(b)** Acrylic top and bottom plates for dynamic test. The bottom plate has ball bearings to reduce friction during oscillatory movement.

A sustained vertical force was applied using a spindle mechanism for the static test. The force was increased from 0 to 80 kg in 2 kg intervals, with each step maintained for 10 seconds to allow the sensor output to stabilize. The frequency response of the timer circuit was recorded at each force level. This setting enabled the maintenance and transmission of the applied force in the opposite direction, amplifying the output force to up to 19560 N (∼2000 kg). We placed each sensor on a fixed acrylic-metal compression test block Fig 3a designed to align the flat surfaces with the anatomical dimensions of the dental arch of the Columbia M-860 dental model.

The dynamic test emulated the oscillatory motion of bruxism by applying a descending force while the test block was suspended on sliding bearings Fig 3b. A motor with a reduction gear induced a circular motion of 1 rev/s (1 Hz), simulating the grinding behavior observed in bruxism. The applied force ranged from 0 to 24 kg in 2 kg intervals, with each test lasting 30 seconds to ensure consistent loading conditions. The frequency response of the timer circuit was recorded continuously, and the average frequency over the 30-second interval was calculated for analysis.

The dynamic test was conducted at room temperature (25°C), and the test duration was limited to prevent significant heat generation and to account for PVDF’s viscoelastic properties. Preliminary tests confirmed that the temperature rise during the 30-second test was negligible (<1°C), ensuring that thermal effects did not influence the results.

Dynamic testing evaluates PVDF membranes under varying conditions, such as changing loads. It involves assessing the sensor’s response to time-varying mechanical stimuli, such as vibrations, impacts, or oscillatory forces. This type of testing is crucial for understanding how the sensor performs in real-world applications where conditions are not static.

**Fig 4:**
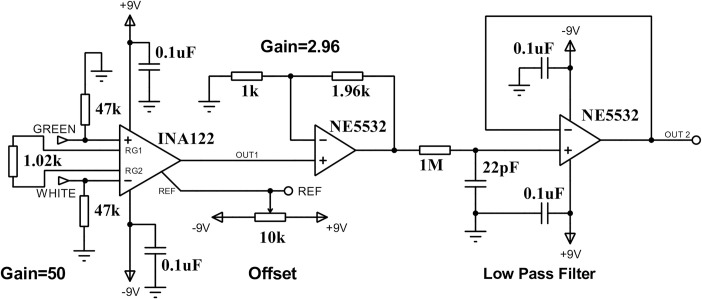
Load cell conditioning circuit. Differential amplifier with a gain of 50. A voltage divider adjusts the signal offset, and then a non-inverter amplifier with a gain of 2.96 connects to a first-order active low-pass filter with a cut-off frequency of 7.23 kHz.

For both tests, the force applied to each sensor is measured using an Iotoclub Type S load cell with a capacity of 300 kg and a sensitivity of 1.9386 mV/V, connected to a conditioning circuit Fig 4 that amplifies, compensates, and filters the signal generated by the load cell.

Applying 12 V to the load cell outputs a voltage given by equation (1).


$$V_{omax300Kg}\;=\;V_{i}\;*\;Sens\;*\;Gain$$ (1)

The load cell has a DC response of 11.4763 mV/kg.

**Fig 5:**
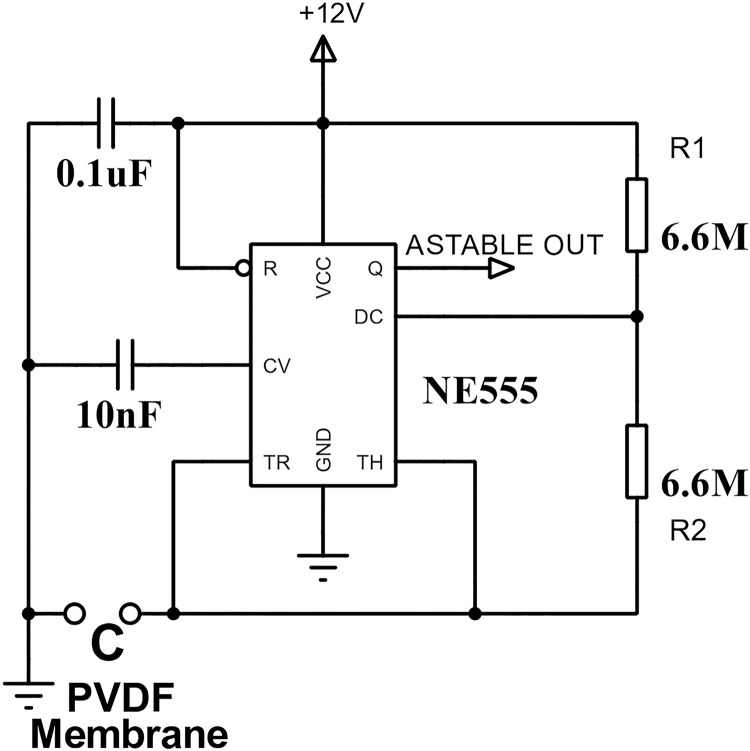
Precision timer circuit in Astable mode operation.


Based on the NE555 integrated circuit, the precision timer circuit operated in Astable mode to generate a pulsed signal Fig 5 to correlate the effect of the descending force applied to the membrane. The RC constant determined the circuit’s output frequency (*f*), which varied with the capacitance of the PVDF membrane. A fixed resistance of 6 MΩ was used to ensure the output signal remained within the nominal operating range of the circuit.

$$f=\frac{1.44}{\left(R_{1}+R_{2}\right)C_{PVDF}}$$ (2)

Before testing, the baseline capacitance of each membrane was measured using a digital LCR bridge. These values were used to calculate the initial output frequency of the timer circuit, as shown in Table 2. Any deviations from the baseline were attributed to the applied force.

**Table 2:** Membrane initial conditions for static and dynamic tests.

Parameter	Static Test		Dynamic Test	
	Sensor A	Sensor B	Sensor A	Sensor B
Capacitance (pF)	23.3	16.1	24.1	16.3
Precision timer output (kHz) Theoretical	3.433	4.969	3.320	4.908
Frequency (kHz) Experimental	3.458	4.935	3.356	4.968
% Error	0.73	0.68	1.08	1.22

The sensors were securely mounted on the test block using adhesive tape to minimize artifacts caused by membrane misalignment, electrode lead movement, and spindle mechanism vibration. The initial force applied to the sensor was adjusted to zero, and baseline capacitance values were measured before each test to ensure consistency.

To ensure reproducibility, two sets of three tests each evaluated the performance of PVDF membranes as force sensors under controlled conditions emulating bruxism behavior. Each set analyzed the frequency response of each membrane to a force sweep from 0 to 80 kg for the static test and from 0 to 24 kg for the dynamic test in 2 kg intervals.

Triplicate static and dynamic loading tests on the PVDF membrane sensor in a simulated bruxism environment strengthen the study’s conclusions. Testing static and dynamic conditions reflects the varied forces involved in bruxism, capturing clenching and grinding behaviors. Three repetitions enhance reliability by accounting for system variability and establishing consistent trends or anomalies in the sensor’s response. This approach balances thoroughness and practicality, offering enough data for statistical significance without excessive resource expenditure.

The standard deviation of the frequency measurements was calculated to assess the consistency of the sensor response. Any outliers were identified and excluded from the analysis.

The Iotoclub Type S load cell was calibrated to ensure the precision and reliability of force measurements throughout the study using known weights (0–300 kg) prior to testing. The load cell was mounted on a stable test platform to minimize external vibrations and misalignments, which could compromise measurement accuracy. The load cell was adjusted to a zero-load condition to eliminate baseline drift and residual signals that could introduce measurement errors. A voltage divider circuit to nullify the offset voltage ensures that the output signal is zero without applied force.

### Linear regression analysis and validation

A linear response in PVDF membranes implies that the capacitance output is directly proportional to the applied mechanical force. This behavior is desirable as it ensures predictable and accurate measurements. Deviations from linearity, such as saturation or hysteresis, can complicate data interpretation and reduce sensor reliability.

A linear regression model was employed to define the relationship between the applied force and the frequency of the precision timer circuit dependent on the sensor capacitance response (equation 2). This model allowed for quantifying the sensor’s sensitivity to express variations in frequency (kHz) to changes in force (kg). The coefficient of determination R² was then calculated to assess this linear model’s overall goodness of fit, representing the proportion of variance in the frequency response explained by the applied force.

The p-value associated with the regression coefficient was calculated to determine the statistical significance of the observed relationship. A small p-value (less than 0.05) indicates a statistically significant relationship between force and frequency response, suggesting that the observed relationship is unlikely due to chance.

The Breusch-Pagan test was used to evaluate the homoscedasticity (constant variance of residuals) assumption to assess the validity of the linear regression model, which requires the variance of the residuals to be constant across all levels of the independent variable (force). Two tests, the Cook-Weisberg and White test, were conducted to evaluate heteroscedasticity (non-constant variance of residuals), which could invalidate the linear model. The White test is more comprehensive and flexible, making it ideal for detecting complex patterns of heteroscedasticity. In contrast, the Cook-Weisberg test is simpler and more straightforward, suitable for general cases [54].

Finally, the Durbin-Watson statistic was calculated to assess the independence of the residuals, checking for autocorrelation, which can also violate the assumptions of linear regression. These statistical tests and graphical analysis of residuals provide a comprehensive evaluation of the linear model’s validity and the sensor’s performance in measuring bite force.

## 2 Results

We obtained frequency measurements for each level of applied force by averaging the three values from each repetition in both the static and dynamic tests.

Table 2 displayed the initial capacitance and frequency values as the baseline conditions.

### 2.1 Static test

**Fig 6:**
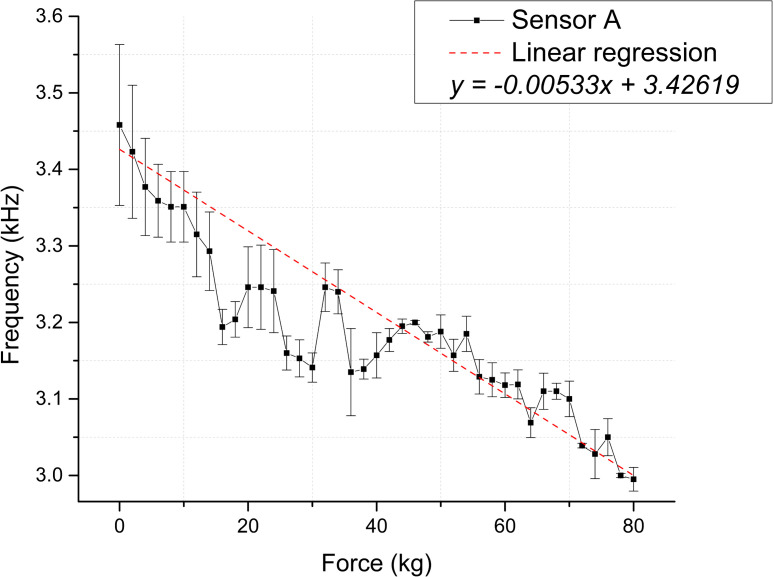
Static test membranes response for Sensor A. Correspondence between applied force and frequency, the dashed line depicts the linear regression S1 Table.

**Fig 7:**
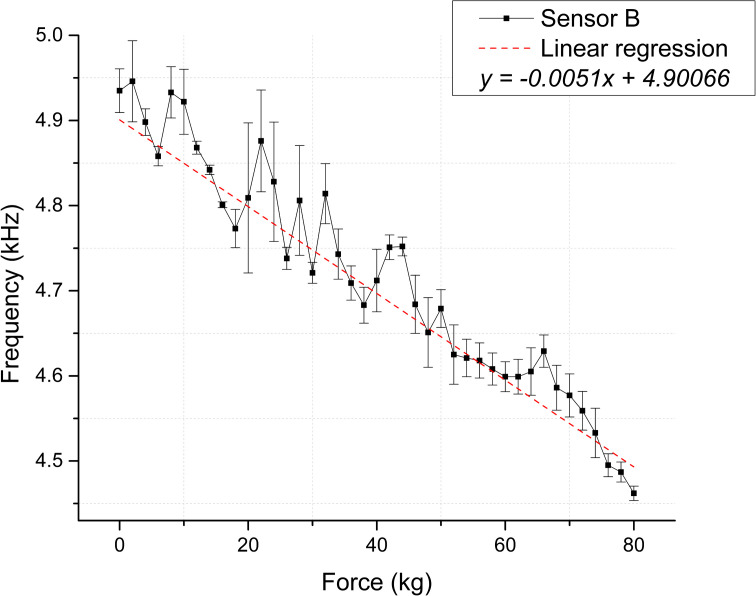
Static test membranes response for Sensor B. Correspondence between applied force and frequency, the dashed line depicts the linear regression S2 Table.

As depicted in Fig 6 and 7, 41 measurements were obtained for each membrane, illustrating the relationship between the applied force and the output frequency of the precision timer circuit. The measurement range was 463 Hz and 484 Hz for Sensors A and B, respectively S1 File.

The experimental data indicates a linear trend between both membranes’ applied force and output frequency. Table 3 presents the statistical values derived from the linear regression analysis to support this correspondence.

**Table 3:** Static test statistics.

Sensor	_R_2	p-Value	BP p-value^1^	CW p-value^2^	W p-value^3^	d static^4^
A	0.91046	1.9593x10^*−*16^	0.018	0.261	0.119	0.901
B	0.94718	2.7168x10^*−*25^	0.407	0.128	0.078	1.529

^1^Breusch-Pagan p-value.

^2^Cook-Weisberg p-value.

^3^White p-value.

^4^Durbin-Watson statistics.

**Fig 8:**
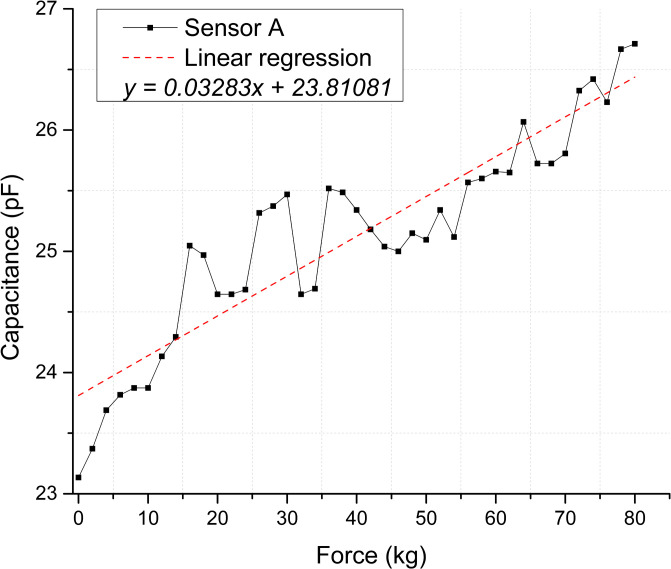
Static test membranes response for Sensor A. Correspondence between applied force and calculated capacitance.

**Fig 9:**
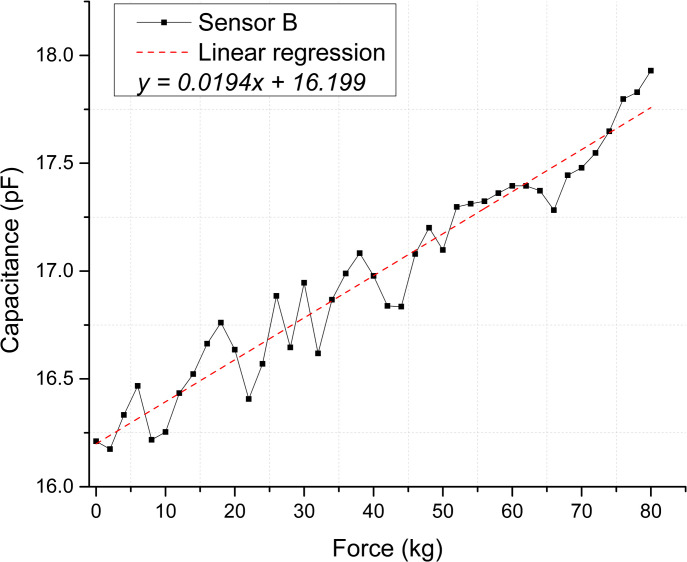
Static test membranes response for Sensor B. Correspondence between applied force and calculated capacitance.

The scatter plots shown in Fig 8 and Fig 9 depict the calculated capacitance values derived from the frequency measurements for each repetition. Based on the linear regression slope, the membranes’ sensitivity can be estimated at 0.03283 pF/kg for Sensor A and 0.01949 pF/kg for Sensor B S3 and S4 Tables.

### 2.2 Dynamic test

**Fig 10:**
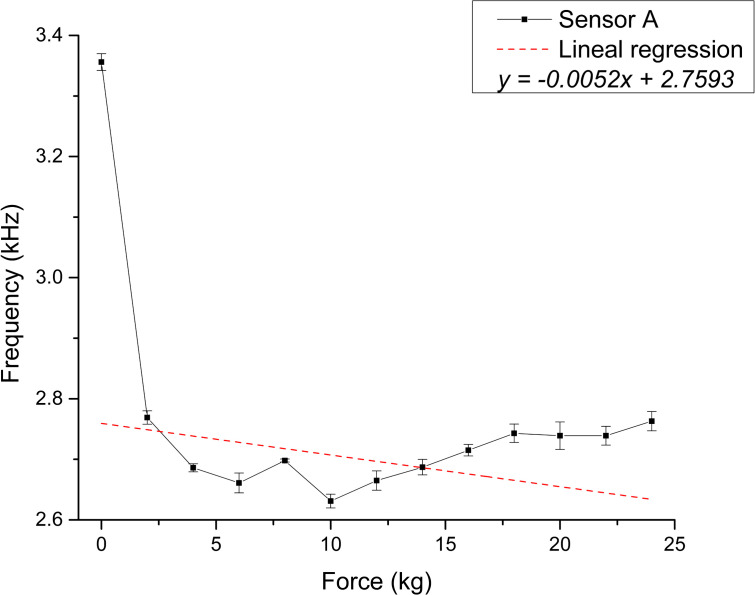
Dynamic test sensor response for Sensor A, depicting the relationship between applied force and output frequency. The dashed line depicts the linear regression S5 Table.

**Fig 11:**
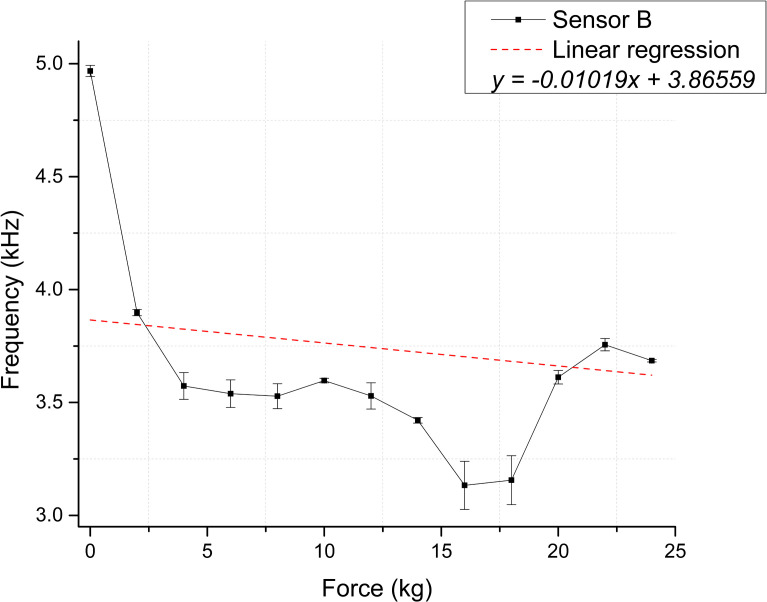
Dynamic test sensor response for Sensor B, depicting the relationship between applied force and output frequency. The dashed line depicts the linear regression S6 Table.

To evaluate the PVDF membranes’ performance under dynamic conditions that simulate the oscillatory behavior of bruxism, we conducted 13 measurements for each membrane. Figs 10 and 11 show how the applied force affects the precision timer circuit’s output frequency. Based on the initial conditions of both membranes, as Table 2 presents, the measurement range was between 593 Hz for Sensor A and 1283 Hz for Sensor B S2 File.

The experimental data of the dynamic tests do not show a trend as linear and pronounced as the one observed in the static tests. Table 4 shows the statistical values from the linear regression analysis to ensure consistency in analyzing results across the two testing modalities.

**Table 4:** Dynamic test statistics.

Sensor	_R_2	p-Value	BP p-value^1^	CW p-value^2^	W p-value^3^	d static^4^
A	−0.0339	0.2181	0.067	0.875	0.065	0.198
B	0.0317	0.0867	0.264	0.767	0.218	0.527

^1^Breusch-Pagan p-value.

^2^Cook-Weisberg p-value.

^3^White p-value.

^4^Durbin-Watson statistics.

**Fig 12:**
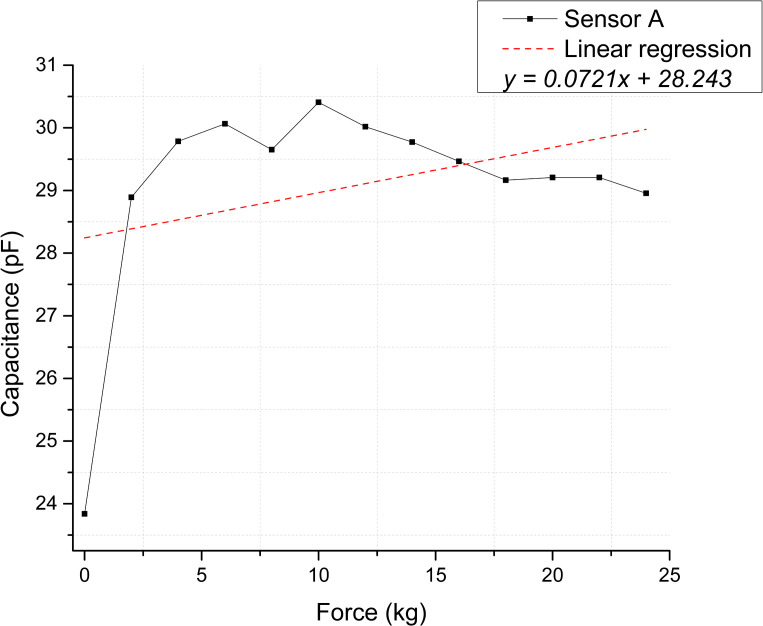
Dynamic test membranes response for Sensor A. Correspondence between applied force and calculated capacitance.

**Fig 13:**
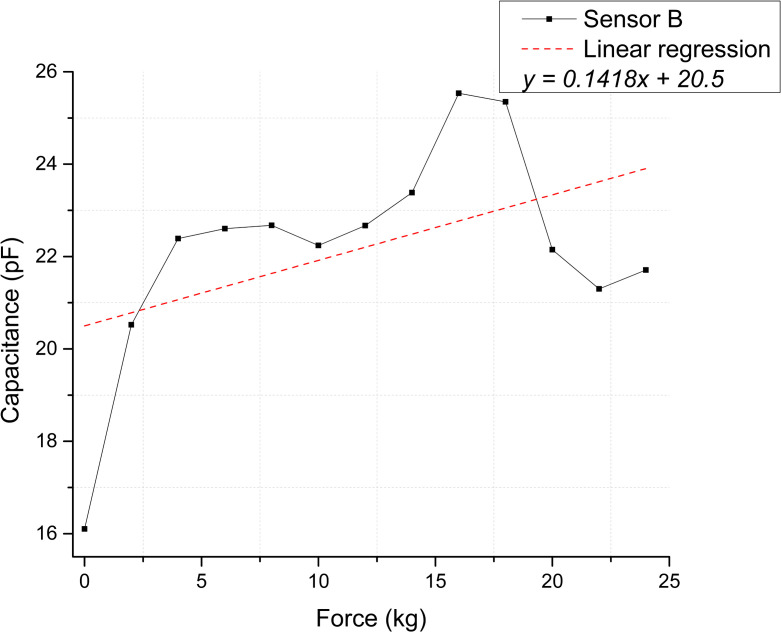
Dynamic test membranes response for Sensor B. Correspondence between applied force and calculated capacitance.

Figs 12 and 13 illustrate the sensor response regarding calculated capacitance derived from the frequency measurements. Based on this conversion, the estimated sensitivities for Sensor A and B are 0.07218 pF/kg and 0.14188 pF/kg, respectively S7 and S8 Tables.

The limited number of samples in the dynamic tests resulted from the mechanical constraints inherent in the instrumental setup developed. Specifically, the mechanism responsible for generating oscillatory motion could maintain a constant speed of 1 revolution per second under loads less than 25 kg.

Furthermore, considering the real-world scenario of a bruxism episode with grinding, the range of bite force typically extends between 30% and 60% of the maximum masticatory force. Therefore, the range of forces employed in the dynamic tests was suitable to simulate oscillatory manifestations of bruxism behavior.

**Fig 14:**
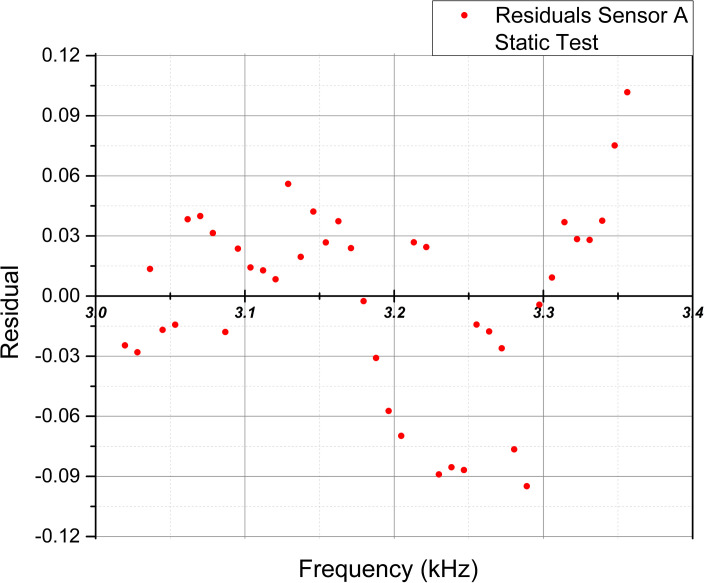
Scatter plot of fitted frequency values [kHz] and residuals for Sensor A extracted from the linear regression analysis in the static test.

**Fig 15:**
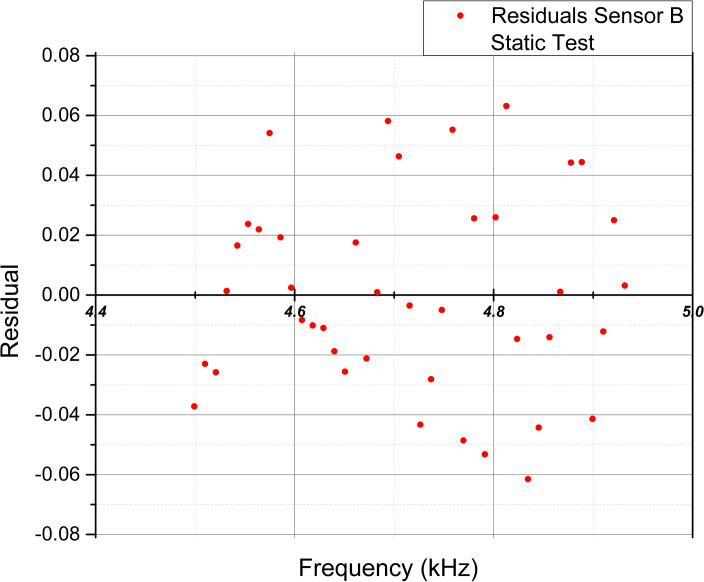
Scatter plot of fitted frequency values [kHz] and residuals for Sensor B extracted from the linear regression analysis in the static test.

**Fig 16:**
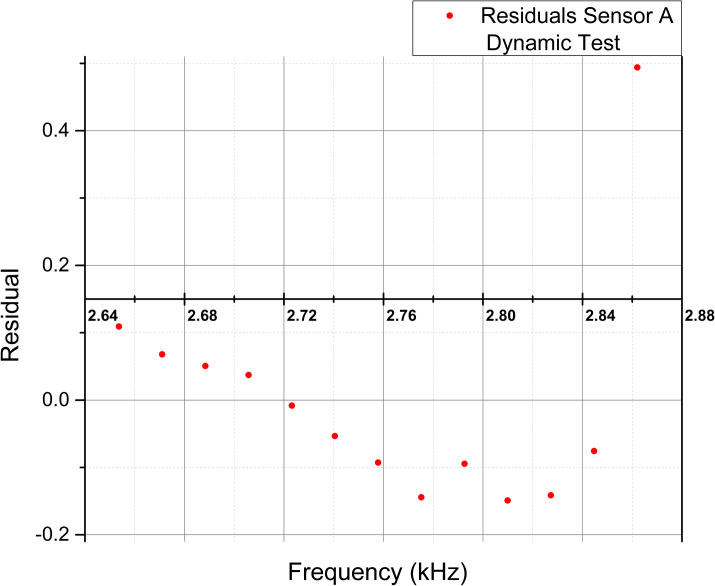
Scatter plot of fitted frequency values [kHz] and residuals for Sensor A extracted from the linear regression analysis in the dynamic test.

**Fig 17:**
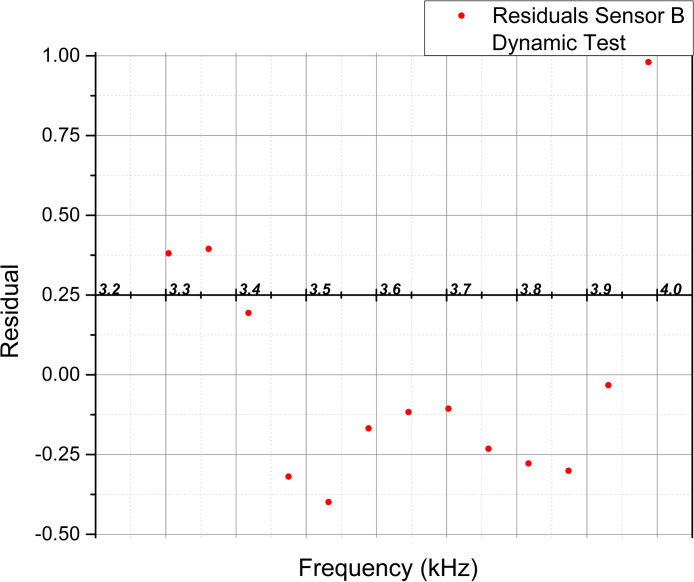
Scatter plot of fitted frequency values [kHz] and residuals for Sensor B extracted from the linear regression analysis in the dynamic test.

To complement the examination of the statistical values, Figs 14–17 show the scatter plots of the fitted values and residuals, which assess the data’s homoscedasticity and heteroscedasticity by the characteristic dispersion pattern.

## 3 Discussion

PVDF membranes represent a significant advancement by offering a more versatile means of measuring sustained force variations, as demonstrated in the study, which evaluated sensor responses to controlled scenarios emulating bruxism behavior. By analyzing the linear trends in measurements across different experiments and conducting statistical analyses to assess the strength of the linear relationship, the validity of the linear model, and the independence of successive measurements, PVDF membranes provide a robust and accurate alternative to conventional techniques, enhancing the ability to monitor and diagnose bruxism-related conditions effectively.

Unlike traditional bruxism assessment methods such as EMG and conventional force sensors, PVDF sensors leverage the piezoelectric effect to conform a force–capacitance–frequency system as a viable alternative for their high sensitivity, flexibility, and ability to measure multiple physiological variables such as force, temperature, or humidity. Their low power consumption and ease of integration into wearable devices make them well-suited for continuous, real-time masticatory monitoring, offering patients a comfortable and non-invasive solution. In contrast, while effective for capturing muscle activity, EMG systems are limited by their susceptibility to interference, electrode displacement, and power requirements, which hinder their use in long-term or sleep monitoring. Piezoresistive sensors, operating on the principle of resistance change under pressure, and piezoelectric sensors which output electric charge in response to mechanical stress, provide reliable force measurements but lack the flexibility, sensitivity, and multifunctionality of PVDF sensors, making them less ideal for advanced wearable applications.

The comparison depicted in Table 5. Instrumental Paradigms in Bruxism Monitoring: EMG/Force Sensors Systems vs. PVDF Sensor Solutions underscores the potential of PVDF sensors to become an instrumental bruxism assessment alternative by addressing the limitations of current technologies and projecting innovative approximations that encompass comprehensive monitoring solutions.

**Table 5:** Instrumental paradigms in Bruxism monitoring: EMG/force sensors systems vs. PVDF sensor solutions.

Feature	PVDF sensors	EMG	Force sensors
Primary Use	Bite force measurement [55–59]	Muscle activity monitoring (masseter, temporalis) [24,60,61]	Bite force measurement [9,35,49,62]
Working Principle	Capacitive response leveraged by piezoelectric effect [2,56]	Measures electrical activity of muscles using surface electrodes [24,60]	Piezoresistive or piezoelectric effect [9,49,63,64]
Hardware Requirements	Low-power signal conditioning circuits, film electrodes [32,56,65]	Signal amplifiers, filters, electrical isolation, specialized electrodes [19,61,66]	Signal conditioning circuits, rigid or semi-flexible substrates [9,37,67]
Output Signal	Capacitance proportional to applied force or vibration [32,56]	Digital voltage proportional to muscle electrical activity [60]	Resistance is inversely proportional to applied pressure. Output charge generated proportionally to the rate of force change [9,32,39]
Flexibility	Highly flexible; conforms to jaw anatomy [32,56,57,59,65]	Limited conformability due to rigid electrodes [60,61]	Limited adaptability to jaw contours [9,49,63,64]
Wearability	Thin, lightweight design facilitate integration into wearable devices [59,65,68,69]	Potential electrode displacement during long-term monitoring [61,70–72]	Challenging to integrate into wearables [37,62,73,74]
Comfort	Suitable for extended use with minimal increase of vertical dimension [58,65]	Potential discomfort from electrodes [52,60,71]	Reduced mandibular mobility due to rigidity. Post use discomfort [9,75,76].
Power Consumption	Low power requirements; enable battery operation [32,56,57]	Higher power demands necessitate frequent recharging [61,70,77]	Moderate to high power efficiency depending on biofeedback stimuli [73,74,76].
Real-Time Monitoring	Wide frequency response with no delay in detecting force changes [2,32,65]	Delayed or offline analysis [61,66,71,72]	Bulkiness may restrict real-time functionality on long-term monitoring [39,73,74]
Durability	High resistant to mechanical wear [68,69,78,79]	Electrode degradation may occur with prolonged use [60,66]	Moderate resistance to wear [9,80,81]
Environmental Stability	Maintains performance across varying conditions (temperature, humidity) [63,64,68]	Susceptible to environmental interference [19,66,72]	Performance may vary under extreme conditions [62,81,82]
Ease of Integration	Straightforward incorporation into wearable designs [32,58,65]	Limited incorporation with dental devices [19,66,72]	Integration challenges due to physical constraints [62,73,74]
Cost	Moderate expense with favorable long-term value [32,56–58]	Higher costs associated with specialized components [19,61]	Moderate expense but less economical for wearable applications over long-term periods [73,74,80]
Measurement of Other Variables	Capable of measuring vibrations, temperature and humidity [2,56,58]	Can be combined with other sensors for physiological monitoring [24,60,72]	Primarily limited to force measurement [9,62,75]
Clinical Feasibility	Requires patient-specific intraoral integration for optimal performance [32,57,58,63,64,79]	Standardized electrode placement protocols exist [19,24,82]	Prefabricated designs limit individual adaptation [61,62,75,76]

### Insights from static and dynamic testing

The coefficient of determination (R²) was used to assess the strength of the linear relationship between variables. Static tests showed strong linear trends, with R² values of 0.79 and 0.92 for Sensors A and B, respectively. The associated p-values (1.9593 × 10 ⁻ ¹⁶ and 2.7168 × 10 ⁻ ²⁵) were well below the significance level, confirming statistically significant linear relationships. In contrast, dynamic tests showed weaker correlations, with low or even negative R² values and p-values exceeding the significance threshold (0.2181 and 0.0867), indicating a poor linear relationship. This discrepancy is likely influenced by the viscoelastic behavior of PVDF under cyclic loading, a characteristic of polymers, which introduces nonlinear responses. Further investigation into PVDF’s dynamic mechanical properties, such as storage and loss moduli, could provide deeper insights into this behavior.

Implementing the Breusch-Pagan test in the Static test, the p-value (0.018) indicated heteroscedasticity for Sensor A, while Sensor B (0.407) and both sensors in the dynamic test (0.067, 0.264) showed no evidence of heteroscedasticity, though homoscedasticity was not confirmed. Residual plots supported these findings, with Sensor A in the static test and both sensors in the dynamic test displaying patterns suggesting heteroscedasticity. Sensor B exhibited a random scatter in the static test, ruling out heteroscedasticity but not confirming homoscedasticity. Notably, the lack of homoscedasticity does not invalidate the linear relationship, as it may arise from an inversely proportional relationship between force and frequency, where error variance is more significant at lower force values.

The Cook-Weisberg and White tests were conducted to further validate the linear model’s assumptions. Both tests yielded p-values greater than 0.05, supporting the absence of heteroscedasticity in most cases and reinforcing the linear model’s robustness for static tests. However, the dynamic tests’ results, combined with the observed autocorrelation and nonlinear behavior, suggest that the linear model may not be suitable for dynamic conditions.

The Durbin-Watson test was employed to detect autocorrelation in residuals, which could affect the validity of statistical inferences. Sensor B in the static test (d-statistic = 1.529) suggested no autocorrelation, while Sensor A in the static test (0.901) and both sensors in the dynamic test (0.875, 0.767) exhibited positive autocorrelation that may be attributed to the material memory or stability of PVDF, which influences the capacitive response under repeated measurements.

In summary, this comprehensive statistical analysis, incorporating R², p-values, the Breusch-Pagan test, the Durbin-Watson test, and the Cook-Weisberg and White tests, thoroughly evaluates the linear trends in the data. While static tests demonstrate strong, statistically significant linear relationships, dynamic tests reveal limitations due to PVDF’s viscoelastic properties and autocorrelation effects. Future research should focus on refining the model to account for these dynamic behaviors and exploring advanced signal processing techniques to improve accuracy in real-world applications.

The statistical analysis presented does not aim to establish a model that definitively describes the behavior of the PVDF sensors designed with the methodology discussed. Instead, it seeks to provide an initial approximation that allows interpreting, based on a simple linear estimation, whether the relationship between the force exerted on the sensor surface and the electrical frequency signal can maintain measurable correspondence to quantify bite force in different scenarios characteristic of bruxism behavior, without reflecting sampling errors, correlated variability, outliers, or trends in variance that could compromise the development and reinforcement of the presented methodology and the proposed adjustment models that would allow effectively estimating the behavior of the membranes.

Exploring nonlinear models, data transformations, and time series modeling techniques to estimate the sensor behavior in each experiment represents an important focus for future work in this team’s ongoing research.

### Broader implications and limitations

The findings of this study highlight the potential of PVDF membranes as a significant advancement over traditional bruxism assessment methods. However, it is important to acknowledge the limitations of the current research. The experiments were conducted under controlled laboratory conditions, which may not fully replicate the complex oral environment encountered during bruxism. Factors such as saliva, temperature fluctuations, and prolonged cyclic loading were not accounted for, potentially limiting the generalizability of the results. It should be noted that clinical trials involving intraoral devices using piezoelectric sensors have already been reported [75,76] demonstrating promising real-world applications for PVDF-based sensors in monitoring bruxism, suggesting that the transition from laboratory testing to clinical implementation exploring the integration of the sensor into wearable devices for real-time bruxism monitoring could provide valuable insights into its practical applicability. Investigating the long-term performance of the sensor, including potential degradation of the PVDF membrane under sustained use, would also be critical for ensuring its reliability in clinical settings. The existing clinical studies provide a strong foundation for such future work, reinforcing the potential of PVDF-based sensors as a viable tool for bruxism assessment.

Beyond bruxism assessment, one area of interest is the development of composite piezoelectric materials. While PVDF is widely used due to its flexibility and biocompatibility, its piezoelectric properties are modest compared to ceramic materials such as PZT. Incorporating nanofillers, such as graphene, carbon nanotubes, or ceramic particles, into PVDF could enhance its piezoelectric performance, mechanical strength, and thermal stability, opening new possibilities for sensor applications. Furthermore, future research should prioritize the development of practical prototypes for specific use cases, such as wearable health monitoring devices, embedded sensors for smart structures, or energy-harvesting systems for low-power electronics. Addressing challenges related to durability, signal processing, and integration with existing technologies will be essential for translating laboratory-scale innovations into real-world solutions.

Another critical area for future investigation is improving the environmental resilience of PVDF sensors. Given their sensitivity to temperature and humidity, exploring protective coatings, encapsulation methods, or material modifications could enhance their performance in harsh or variable conditions. Additionally, advanced signal processing techniques, including noise reduction algorithms, signal amplification, and real-time data analysis, could significantly improve the accuracy and usability of PVDF sensors. Finally, research into scalable and cost-effective manufacturing methods for PVDF sensors could facilitate their widespread adoption. Investigating techniques for film production, electrode deposition, and sensor assembly that balance performance and affordability will be crucial for expanding their applications across medical, industrial, and consumer domains.

### Advancing bruxism assessment

The manuscript highlights the potential of PVDF sensors for bruxism detection but lacks explicit details on differentiating bruxism from normal occlusion. To address this, we elucidate an innovative framework that leverages PVDF sensors’ piezoelectric properties to measure force-induced capacitance variations, visualized as frequency signals through a timer circuit. This approach enables the identification of unique frequency signatures associated with bruxism, such as irregular, high-force patterns, distinguishing them from the controlled forces of normal occlusion where baseline data from normal occlusion activities and bruxism cases are used to develop algorithms, incorporating machine learning or threshold-based systems for classification enhancing the interpretation of sensor data in complex environments, enabling more precise and reliable measurements.

Practical considerations such as user comfort, device miniaturization, and data privacy must be addressed to ensure such devices’ feasibility and ethical use. By adopting this framework, PVDF sensor measurements can be effectively translated into a reliable assessment tool for bruxism, offering a clear distinction between bruxism and normal occlusion based on quantifiable differences in force and frequency output patterns. This innovative approach has the potential to significantly advance the field of bruxism diagnostics and improve patient outcomes.

## 4 Conclusion

This study investigated the response capabilities of a PVDF membrane force sensor by examining the relationship between applied force and the sensor’s capacitive output, measured through frequency response using a precision timer circuit. The results demonstrate that PVDF membranes have significant potential for quantifying bite force, particularly in research on understanding bruxism behavior. However, further research is needed to explore alternative sensor designs and advanced polarization techniques, such as electrospinning, to enhance the proposed methodology’s sensitivity, accuracy, and overall performance.

PVDF membranes offer a promising alternative for bruxism assessment, addressing critical limitations of current methods in bite force measurement and opening new possibilities for both research and clinical applications. Unlike traditional approaches, which are often subjective, invasive, costly, and limited in their ability to provide continuous monitoring, PVDF sensors enable objective, noninvasive, cost-effective, and real-time measurement of bite force. This innovative approach has the potential to transform our understanding of bruxism and refine treatment strategies by providing reliable, continuous data in natural settings. Efforts will focus on optimizing sensor sensitivity, enhancing biocompatibility, and refining fabrication techniques to broaden the utility of PVDF membranes in dentistry and biomedicine, ultimately advancing their clinical adoption and impact.

Beyond bruxism, PVDF membranes show promise for a range of dental applications, such as monitoring orthodontic forces and detecting forces in multiple directions, optimizing prosthodontic treatments; they also can be integrated into dental devices to harvest energy from mechanical movements (e.g., chewing) to power low-energy dental sensors or diagnostic tools. Their versatility makes them a valuable tool for advancing dental diagnostics and treatment. This study underscores the importance of recognizing bruxism as a multifactorial phenomenon and highlights the need for adaptable, innovative evaluation techniques to drive progress in research and clinical practice. By continuing to refine and expand the capabilities of PVDF-based sensors, we can pave the way for more effective, personalized, and accessible solutions for bruxism and related conditions, improving patient outcomes and advancing the field of dental medicine [83-92].

## Supporting information

S1 File DATA static test. (XLSX)

S2 File DATA dynamic test. (XLSX)

S1 Table Static sensor_a. (PDF)

S2 Table Static sensor_b. (PDF)

S3 Table Static sensor_a capacitance. (PDF)

S4 Table Static sensor_b capacitance. (PDF)

S5 Table Dynamic sensor_a. (PDF)

S6 Table Dynamic sensor_b. (PDF)

S7 Table Dynamic sensor_a capacitance. (PDF)

S8 Table Dynamic sensor_b capacitance. (PDF)

## References

[1] Dohany JE. Fluorine‐containing polymers, Poly(Vinylidene Fluoride). Kirk-othmer encyclopedia of chemical technology. Wiley; 2000. doi: 10.1002/0471238961.1615122504150801.a01

[2] Concha VOC, Timóteo L, Duarte LAN, Bahú JO, Munoz FL, Silva AP, et al. Properties, characterization and biomedical applications of polyvinylidene fluoride (PVDF): a review. J Mater Sci. 2024;59(31):14185–204. doi: 10.1007/s10853-024-10046-3

[3] Martins P, Lopes AC, Lanceros-Mendez S. Electroactive phases of poly(vinylidene fluoride): determination, processing and applications. Prog Polymer Sci. 2014;39(4):683–706. doi: 10.1016/j.progpolymsci.2013.07.006

[4] Das-Gupta DK, Doughty K, Shier DB. A study of structural and electrical properties of stretched polyvinylidene fluoride films. J Electrostatics. 1979;7:267–82. doi: 10.1016/0304-3886(79)90079-2

[5] Shehata N, Nair R, Boualayan R, Kandas I, Masrani A, Elnabawy E, et al. Stretchable nanofibers of polyvinylidenefluoride (PVDF)/thermoplastic polyurethane (TPU) nanocomposite to support piezoelectric response via mechanical elasticity. Sci Rep. 2022;12(1):8335. doi: 10.1038/s41598-022-11465-5 35585095 PMC9117269

[6] Nivedhitha DM, Jeyanthi S. Polyvinylidene fluoride, an advanced futuristic smart polymer material: a comprehensive review. Pol Adv Techs. 2022;34(2):474–505. doi: 10.1002/pat.5914

[7] Hernández-Rivera D, Rodríguez-Roldán G, Mora-Martínez R, Suaste-Gómez E. A capacitive humidity sensor based on an electrospun PVDF/Graphene membrane. Sensors (Basel). 2017;17(5):1009. doi: 10.3390/s17051009 28467357 PMC5469532

[8] Haghiashtiani G, Greminger M. Fabrication, polarization, and characterization of PVDF matrix composites for integrated structural load sensing. Smart Mater Struct. 2015;24.

[9] González-Morán CO, Pérez GAZ, Suaste-Gómez E. System for controlling the moisture of the soil using humidity sensors from a polyvinylidenefluoride fiber mats. Adv Sci Lett. 2013;19(3):858–61. doi: 10.1166/asl.2013.4869

[10] Suaste-Gómez E, Rodríguez-Roldán G, Reyes-Cruz H, Terán-Jiménez O. Polymeric prosthesis as acoustic, pressure, temperature, and light sensor fabricated by three-dimensional printing. In: Toshio O ed. Piezoelectric materials. Rijeka: InTech; 2016. doi: 10.5772/63074

[11] Bae J-H, Chang S-H. PVDF-based ferroelectric polymers and dielectric elastomers for sensor and actuator applications: a review. Funct Compos Struct. 2019;1(1):012003. doi: 10.1088/2631-6331/ab0f48

[12] Ting Y, Suprapto S, Nugraha A, Chiu C-W, Gunawan H. Design and characterization of one-layer PVDF thin film for a 3D force sensor. Sensors and Actuators A: Physical. 2016;250:129–37. doi: 10.1016/j.sna.2016.09.025

[13] Suaste-Gómez E, Rodríguez-Roldán G, Reyes-Cruz H, Terán-Jiménez O. Developing an ear prosthesis fabricated in polyvinylidene fluoride by a 3D printer with sensory intrinsic properties of pressure and temperature. Sensors (Basel). 2016;16(3):332. doi: 10.3390/s16030332 26959026 PMC4813907

[14] Bauer S, Bauer-Gogonea S, Graz I, Kaltenbrunner M, Keplinger C, Schwödiauer R. 25th anniversary article: a soft future: from robots and sensor skin to energy harvesters. Adv Mater. 2014;26(1):149–61. doi: 10.1002/adma.201303349 24307641 PMC4240516

[15] Cheng Z, Zhang Q, Su J, Tahchi ME. Electropolymers for mechatronics and artificial muscles. In: Piezoelectric and acoustic materials for transducer applications. Springer US. 2008. 131–59. doi: 10.1007/978-0-387-76540-27

[16] Martinez RM, Navarro JDC, Gomez VL, Suaste Gomez E. Humidity sensor to support dry eye diagnosis based on polymeric PVDF transfer membrane. IEEE Sensors J. 2022;22(20):19965–74. doi: 10.1109/jsen.2022.3202406

[17] Morán COG, Ballesteros RG, Guzmán MDAR, Gómez ES. Polyvinylidene flouride polymer applied in an intraocular pressure sensor. Jpn J Appl Phys. 2005;44(6L):L885. doi: 10.1143/jjap.44.l885

[18] Yiyang L, Peng Y, Yuechao W, Zaili D, Ning X. The modeling and experiments of a PVDF mirco-force sensor. In: 2008 3rd IEEE International Conference on Nano/Micro Engineered and Molecular Systems, 2008. 60–4. doi: 10.1109/nems.2008.4484286

[19] Mullaveettil FN, Dauksevicius R, Wakjira Y. Strength and elastic properties of 3D printed PVDF-based parts for lightweight biomedical applications. J Mech Behav Biomed Mater. 2021;120:104603. doi: 10.1016/j.jmbbm.2021.104603 34051693

[20] Mathers WD, Binarao G, Petroll M. Ocular water evaporation and the dry eye: a new measuring device. Cornea. 1993;12. https://journals.lww.com/corneajrnl/fulltext/1993/07000/ocularwaterevaporationandthedryeyeanew.10.aspx 10.1097/00003226-199307000-00010 8339563

[21] Cui X, Huang F, Zhang X, Song P, Zheng H, Chevali V, et al. Flexible pressure sensors via engineering microstructures for wearable human-machine interaction and health monitoring applications. iScience. 2022;25(4):104148. doi: 10.1016/j.isci.2022.104148 35402860 PMC8991382

[22] Jacob J, More N, Kalia K, Kapusetti G. Piezoelectric smart biomaterials for bone and cartilage tissue engineering. Inflamm Regen. 2018;38:2. doi: 10.1186/s41232-018-0059-8 29497465 PMC5828134

[23] Wang YR, Zheng JM, Ren GY, Zhang PH. A flexible piezoelectric force sensor based on PVDF fabrics. Smart Mater Struct. 2011;20.

[24] Takeuchi H, Ikeda T, Clark GT. A piezoelectric film-based intrasplint detection method for bruxism. J Prosthet Dent. 2001;86(2):195–202. doi: 10.1067/mpr.2001.115487 11514809

[25] Martin LW, Rappe AM. Thin-film ferroelectric materials and their applications. Nat Rev Mater. 2016;2(2). doi: 10.1038/natrevmats.2016.87

[26] Dargahi J, Najarian S. Human tactile perception as a standard for artificial tactile sensing--a review. Int J Med Robot. 2004;1(1):23–35. doi: 10.1002/rcs.3 17520594

[27] Ramadan KS, Sameoto D, Evoy S. A review of piezoelectric polymers as functional materials for electromechanical transducers. Smart Mater Struct. 2014;23(3):033001. doi: 10.1088/0964-1726/23/3/033001

[28] Lavigne GJ, Rompré PH, Montplaisir JY. Sleep bruxism: validity of clinical research diagnostic criteria in a controlled polysomnographic study. J Dent Res. 1996;75(1):546–52. doi: 10.1177/00220345960750010601 8655758

[29] Kato T, Thie NM, Huynh N, Miyawaki S, Lavigne GJ. Topical review: sleep bruxism and the role of peripheral sensory influences. J Orofac Pain. 2003;17(3):191–213. 14520766

[30] Colonna A, Segù M, Lombardo L, Manfredini D. Frequency of sleep bruxism behaviors in healthy young adults over a four-night recording span in the home environment. Applied Sciences. 2020;11(1):195. doi: 10.3390/app11010195

[31] Firmino MS, Costa CM, Sencadas V, Serrado NJ, Costa P, Gregorio R, et al. Effect of the ceramic grain size and concentration on the dynamical mechanical and dielectric behavior of poly(vinilidene fluoride)/Pb(Zr0.53Ti0.47)O3 composites. Applied Physics A. 2009;96: 899–908. doi: 10.1007/s00339-009-5141-2

[32] Castroflorio T, Mesin L, Tartaglia GM, Sforza C, Farina D. Use of electromyographic and electrocardiographic signals to detect sleep bruxism episodes in a natural environment. IEEE J Biomed Health Inform. 2013;17(6):994–1001. doi: 10.1109/JBHI.2013.2274532 24240717

[33] Manfredini D, Winocur E, Guarda-Nardini L, Paesani D, Lobbezoo F. Epidemiology of bruxism in adults: a systematic review of the literature. J Orofac Pain. 2013;27(2):99–110. doi: 10.11607/jop.921 23630682

[34] De Leeuw R, Klassner GD. Guidelines for assessment, diagnosis, and management orofacial pain. 6th ed. American Academy of Orofacial Pain; 2020.

[35] Lobbezoo F, Ahlberg J, Raphael KG, Wetselaar P, Glaros AG, Kato T, et al. International consensus on the assessment of bruxism: report of a work in progress. J Oral Rehabil. 2018;45(11):837–44. doi: 10.1111/joor.12663 29926505 PMC6287494

[36] Raphael KG, Janal MN, Sirois DA, Dubrovsky B, Wigren PE, Klausner JJ, et al. Masticatory muscle sleep background electromyographic activity is elevated in myofascial temporomandibular disorder patients. J Oral Rehabil. 2013;40(12):883–91. doi: 10.1111/joor.12112 24237356 PMC3889636

[37] Stuginski-Barbosa J, Porporatti AL, Costa YM, Svensson P, Conti PCR. Agreement of the international classification of sleep disorders criteria with polysomnography for sleep bruxism diagnosis: a preliminary study. J Prosthet Dent. 2017;117(1):61–6. doi: 10.1016/j.prosdent.2016.01.035 27460312

[38] Manfredini D, Ahlberg J, Aarab G, Bender S, Bracci A, Cistulli PA, et al. Standardised tool for the assessment of bruxism. J Oral Rehabil. 2024;51(1):29–58. doi: 10.1111/joor.13411 36597658

[39] Palinkas M, Bataglion C, de Luca Canto G, Machado Camolezi N, Theodoro GT, Siéssere S, et al. Impact of sleep bruxism on masseter and temporalis muscles and bite force. Cranio. 2016;34(5):309–15. doi: 10.1080/08869634.2015.1106811 27077268

[40] Ribeiro AB, Pita MS, Ribeiro AB, Garcia AR, Junqueira Zuim PR. Effect of short-term increase in occlusal vertical dimension on masticatory muscle electrical activities and pressure-to-pain threshold: a crossover clinical study. J Prosthet Dent. 2022;128(5):970–6. doi: 10.1016/j.prosdent.2021.01.023 33678437

[41] Manfredini D, Ahlberg J, Castroflorio T, Poggio CE, Guarda-Nardini L, Lobbezoo F. Diagnostic accuracy of portable instrumental devices to measure sleep bruxism: a systematic literature review of polysomnographic studies. J Oral Rehabil. 2014;41(11):836–42. doi: 10.1111/joor.12207 25040303

[42] Yue G, Fuglevand AJ, Nordstrom MA, Enoka RM. Limitations of the surface electromyography technique for estimating motor unit synchronization. Biol Cybern. 1995;73(3):223–33. doi: 10.1007/BF00201424 7548311

[43] Vaughn BV, Giallanza P. Technical review of polysomnography. Chest. 2008;134(6):1310–9. doi: 10.1378/chest.08-0812 19059962

[44] Rossetti LMN, Rossetti PHO, Conti PCR, de Araujo C dos RP. Association between sleep bruxism and temporomandibular disorders: a polysomnographic pilot study. Cranio. 2008;26(1):16–24. doi: 10.1179/crn.2008.004 18290521

[45] Paesani DA, Lobbezoo F, Gelos C, Guarda-Nardini L, Ahlberg J, Manfredini D. Correlation between self-reported and clinically based diagnoses of bruxism in temporomandibular disorders patients. J Oral Rehabil. 2013;40(11):803–9. doi: 10.1111/joor.12101 24112029

[46] Manfredini D, Ahlberg J, Wetselaar P, Svensson P, Lobbezoo F. The bruxism construct: from cut-off points to a continuum spectrum. J Oral Rehabil. 2019;46(11):991–7. doi: 10.1111/joor.12833 31264730

[47] Nishigawa K, Bando E, Nakano M. Quantitative study of bite force during sleep associated bruxism. J Oral Rehabil. 2001;28(5):485–91. doi: 10.1046/j.1365-2842.2001.00692.x 11380790

[48] Tortopidis D, Lyons MF, Baxendale RH, Gilmour WH. The variability of bite force measurement between sessions, in different positions within the dental arch. J Oral Rehabil. 1998;25(9):681–6. doi: 10.1046/j.1365-2842.1998.00293.x 9758398

[49] Verma TP, Kumathalli KI, Jain V, Kumar R. Bite force recording devices - a review. J Clin Diagn Res. 2017;11(9):ZE01–5. doi: 10.7860/JCDR/2017/27379.10450 29207848 PMC5713870

[50] Padma S, Umesh S, Asokan S, Srinivas T. Bite force measurement based on fiber Bragg grating sensor. J Biomed Opt. 2017;22(10):1–6. doi: 10.1117/1.JBO.22.10.107002 29090535

[51] Pollis M, Maoddi P, Letizia M, Manfredini D. Customized appliance device for force detection in bruxism individuals: an observational study. Int J Dent. 2022;2022:2524327. doi: 10.1155/2022/2524327 35747202 PMC9213119

[52] Shimada A, Baad-Hansen L, Svensson P. Effect of experimental jaw muscle pain on dynamic bite force during mastication. Arch Oral Biol. 2015;60(2):256–66. doi: 10.1016/j.archoralbio.2014.11.001 25463903

[53] Röhrle O, Saini H, Ackland DC. Occlusal loading during biting from an experimental and simulation point of view. Dent Mater. 2018;34(1):58–68. doi: 10.1016/j.dental.2017.09.005 29017762

[54] Lobbezoo F, Ahlberg J, Glaros AG, Kato T, Koyano K, Lavigne GJ, et al. Bruxism defined and graded: an international consensus. J Oral Rehabil. 2013;40(1):2–4. doi: 10.1111/joor.12011 23121262

[55] Sateia MJ. International classification of sleep disorders-third edition: highlights and modifications. Chest. 2014;146(5):1387–94. doi: 10.1378/chest.14-0970 25367475

[56] D. KP, Swaminathan AA, D. AP. A review of current concepts in bruxism – diagnosis and management. J Health Allied Sci NU. 2014;04(04):129–36. doi: 10.1055/s-0040-1703852

[57] Klasser GD, Rei N, Lavigne GJ. Sleep bruxism etiology: the evolution of a changing paradigm. J Can Dent Assoc. 2015;81:f2. 25633110

[58] Koc D, Dogan A, Bek B. Bite force and influential factors on bite force measurements: a literature review. Eur J Dent. 2010;4(2):223–32. 20396457 PMC2853825

[59] Bakke M. Bite force and occlusion. Semin Orthod. 2006;12(2):120–6. doi: 10.1053/j.sodo.2006.01.005

[60] Calderon P dos S, Kogawa EM, Lauris JRP, Conti PCR. The influence of gender and bruxism on the human maximum bite force. J Appl Oral Sci. 2006;14(6):448–53. doi: 10.1590/s1678-77572006000600011 19089246 PMC4327298

[61] Shushma G, Kantly R. Analysis of maximum biting force using gnathodynamometer in different age groups. J Evol Med Dental Sci. 2016;5(100):7323–6. doi: 10.14260/jemds/2016/1658

[62] Revilla-León M, Kois DE, Zeitler JM, Att W, Kois JC. An overview of the digital occlusion technologies: Intraoral scanners, jaw tracking systems, and computerized occlusal analysis devices. J Esthet Restor Dent. 2023;35(5):735–44. doi: 10.1111/jerd.13044 37021739

[63] Escoto-Mora G, González-Morán CO, Suaste-Gómez E. Development of Poly(vinylidene flouride) polymer applied in force sensors for gait analysis in wistar mice of physiology research laboratory. Jpn J Appl Phys. 2008;47(6R):4769. doi: 10.1143/jjap.47.4769

[64] Yachida W, Arima T, Castrillon EE, Baad-Hansen L, Ohata N, Svensson P. Diagnostic validity of self-reported measures of sleep bruxism using an ambulatory single-channel EMG device. J Prosthodont Res. 2016;60(4):250–7. doi: 10.1016/j.jpor.2016.01.001 26876908

[65] Wetselaar P, Manfredini D, Ahlberg J, Johansson A, Aarab G, Papagianni CE, et al. Associations between tooth wear and dental sleep disorders: a narrative overview. J Oral Rehabil. 2019;46(8):765–75. doi: 10.1111/joor.12807 31038764 PMC6852513

[66] Wooldridge JM. Introductory econometrics: a modern approach. 5th ed. South-Western Cengage Learning; 2013.

[67] Wang ZL, Song J. Piezoelectric nanogenerators based on zinc oxide nanowire arrays. Science. 2006;312(5771):242–6. doi: 10.1126/science.1124005 16614215

[68] Zhu Y, Guo S, Ravichandran D, Ramanathan A, Sobczak MT, Sacco AF, et al. 3D-printed polymeric biomaterials for health applications. Adv Healthc Mater. 2025;14(1):e2402571. doi: 10.1002/adhm.202402571 39498750 PMC11694096

[69] Linh VTN, Han S, Koh E, Kim S, Jung HS, Koo J. Advances in wearable electronics for monitoring human organs: bridging external and internal health assessments. Biomaterials. 2025;314:122865. doi: 10.1016/j.biomaterials.2024.122865 39357153

[70] Zeng K, Lin Y, Liu S, Wang Z, Guo L. Applications of piezoelectric biomaterials in dental treatments: a review of recent advancements and future prospects. Mater Today Bio. 2024;29:101288. doi: 10.1016/j.mtbio.2024.101288 40018432 PMC11866170

[71] Lavigne GJ, Khoury S, Abe S, Yamaguchi T, Raphael K. Bruxism physiology and pathology: an overview for clinicians. J Oral Rehabil. 2008;35(7):476–94. doi: 10.1111/j.1365-2842.2008.01881.x 18557915

[72] Yamaguchi T, Mikami S, Maeda M, Saito T, Nakajima T, Yachida W, et al. Portable and wearable electromyographic devices for the assessment of sleep bruxism and awake bruxism: a literature review. CRANIO®. 2020;41(1):69–77. doi: 10.1080/08869634.2020.1815392 32870753

[73] Ardila C-M, Jiménez-Arbeláez GA, Vivares-Builes AM. Efficacy of wireless sensors in assessing occlusal and bite forces: a systematic review. J Oral Rehabil. 2024;51(7):1337–47. doi: 10.1111/joor.13700 38616519

[74] Chen Y, Zhang X, Lu C. Flexible piezoelectric materials and strain sensors for wearable electronics and artificial intelligence applications. Chem Sci. 2024;15: 16436–66. doi: 10.1039/d4sc05166a 39355228 PMC11440360

[75] Zhang J, Wang J, Zhong C, Zhang Y, Qiu Y, Qin L. Flexible electronics: advancements and applications of flexible piezoelectric composites in modern sensing technologies. Micromachines (Basel). 2024;15(8):982. doi: 10.3390/mi15080982 39203633 PMC11356236

[76] Wang ZL, Wang X, Song J, Liu J, Gao Y. Piezoelectric nanogenerators for self-powered nanodevices. IEEE Pervasive Comput. 2008;7(1):49–55. doi: 10.1109/mprv.2008.14

[77] Thymi M, Lobbezoo F, Aarab G, Ahlberg J, Baba K, Carra MC, et al. Signal acquisition and analysis of ambulatory electromyographic recordings for the assessment of sleep bruxism: a scoping review. J Oral Rehabil. 2021;48(7):846–71. doi: 10.1111/joor.13170 33772835 PMC9292505

[78] Jauregi M, Amezua X, Iturrate M, Solaberrieta E. Improving the precision of recordings acquired with digital occlusal analyzers: a dental technique. J Prosthet Dent. 2024;132(1):37–41. doi: 10.1016/j.prosdent.2023.08.001 37661547

[79] Dong W, Xiao L, Hu W, Zhu C, Huang Y, Yin Z. Wearable human–machine interface based on PVDF piezoelectric sensor. Trans Inst Measure Control. 2016;39(4):398–403. doi: 10.1177/0142331216672918

[80] Saito M, Yamaguchi T, Mikami S, Watanabe K, Gotouda A, Okada K, et al. Weak association between sleep bruxism and obstructive sleep apnea. A sleep laboratory study. Sleep Breath. 2016;20(2):703–9. doi: 10.1007/s11325-015-1284-x 26564168

[81] Jadidi F, Castrillon E, Svensson P. Effect of conditioning electrical stimuli on temporalis electromyographic activity during sleep. J Oral Rehabil. 2008;35(3):171–83. doi: 10.1111/j.1365-2842.2007.01781.x 18254794

[82] Li C, Yap S, Loh A, Yap YJ, Kujan O, Balasubramaniam R. Ambulatory devices to detect sleep bruxism: a narrative review. Aust Dent J. 2024;69 Suppl 1(Suppl 1):S53–62. doi: 10.1111/adj.13057 39976111 PMC11937739

[83] Kinjo R, Wada T, Churei H, Ohmi T, Hayashi K, Yagishita K, et al. Development of a wearable mouth guard device for monitoring teeth clenching during exercise. Sensors (Basel). 2021;21(4):1503. doi: 10.3390/s21041503 33671506 PMC7926888

[84] Kim JH, McAuliffe P, O’Connell B, Diamond D, Lau KT. Development of wireless bruxism monitoring device based on pressure-sensitive polymer composite. Sensors and Actuators A: Physical. 2010;163(2):486–92. doi: 10.1016/j.sna.2010.08.033

[85] Aoki R, Takaba M, Abe Y, Nakazato Y, Ohara H, Maejima K, et al. A pilot study to test the validity of a piezoelectric intra-splint force detector for monitoring of sleep bruxism in comparison to portable polysomnography. J Oral Sci. 2022;64(1):63–8. doi: 10.2334/josnusd.21-0421 34955491

[86] Ohara H, Takaba M, Abe Y, Nakazato Y, Aoki R, Yoshida Y, et al. Effects of vibratory feedback stimuli through an oral appliance on sleep bruxism: a 6-week intervention trial. Sleep Breath. 2022;26(2):949–57. doi: 10.1007/s11325-021-02460-7 34370185

[87] Maeda M, Yamaguchi T, Mikami S, Yachida W, Saito T, Sakuma T, et al. Validity of single-channel masseteric electromyography by using an ultraminiature wearable electromyographic device for diagnosis of sleep bruxism. J Prosthodont Res. 2020;64(1):90–7. doi: 10.1016/j.jpor.2019.04.003 31085074

[88] Qi F, Xu L, He Y, Yan H, Liu H. PVDF‐based flexible piezoelectric tactile sensors: review. Cryst Res Technol. 2023;58(10). doi: 10.1002/crat.202300119

[89] Hu Y, Kang W, Fang Y, Xie L, Qiu L, Jin T. Piezoelectric Poly(vinylidene fluoride) (PVDF) polymer-based sensor for wrist motion signal detection. Appl Sci. 2018;8(5):836. doi: 10.3390/app8050836

[90] Jauregi M, Amezua X, Iturrate M, Solaberrieta E. Repeatability and reproducibility of 2 digital occlusal analyzers for measuring the right- and left-side balance of occlusal contact forces: an in vitro study. J Prosthet Dent. 2024;132(1):179–87. doi: 10.1016/j.prosdent.2023.07.026 37661548

[91] Rubió-Ferrer G, Rovira-Lastra B, Khoury-Ribas L, Flores-Orozco EI, Ayuso-Montero R, Martinez-Gomis J. Reference values and reliability of occlusal force distribution and occlusal time measured by the T-Scan system in adults with healthy dentition. J Prosthodont. 2024;33(6):558–64. doi: 10.1111/jopr.13838 38469973

[92] Ohmure H, Oikawa K, Kanematsu K, Saito Y, Yamamoto T, Nagahama H, et al. Influence of experimental esophageal acidification on sleep bruxism: a randomized trial. J Dent Res. 2011;90(5):665–71. doi: 10.1177/0022034510393516 21248360

