# Supplementary material for: Flexible PVDF sensors for bruxism bite force measurement: A redefined instrumental approach
Source: PLoS One. 2025 Aug 21;20(8):e0330422. doi: 10.1371/journal.pone.0330422 (PMC12370117; doi:10.1371/journal.pone.0330422)

Linear Fit (18/10/2024 15:13:17)

Parameters

		Value	Standard Error
Frequency	Intercept	3.42619	0.01661
	Slope	-0.00533	2.63844E-4

Statistics

	Frequency
Number of Points	41
Degrees of Freedom	39
Residual Sum of Squares	340.72246
Pearson's r	-0.95535
Adj. R-Square	0.91046

Summary

	Intercept		Slope		Statistics
	Value	Standard Error	Value	Standard Error	Adj. R-Square
Frequency	3.42619	0.01661	-0.00533	2.63844E-4	0.91046

ANOVA

		DF	Sum of Squares	Mean Square	F Value	Prob>F
Frequency	Model	1	3562.08578	3562.08578	407.72582	0
	Error	39	340.72246	8.73647		
	Total	40	3902.80825			

At the 0.05 level, the slope is significantly different from zero.

Fitted Curves Plot

Frequency

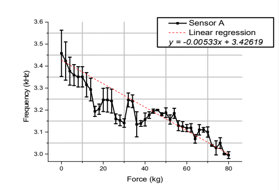

Residual vs. Independent Plot

Frequency

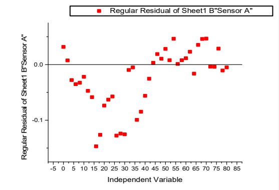

Supplement: S1 Table — (PDF) [file pone.0330422.s003.pdf]
