# Supplementary material for: Flexible PVDF sensors for bruxism bite force measurement: A redefined instrumental approach
Source: PLoS One. 2025 Aug 21;20(8):e0330422. doi: 10.1371/journal.pone.0330422 (PMC12370117; doi:10.1371/journal.pone.0330422)

Linear Fit (30/10/2024 13:13:51)

Parameters

		Value	Standard Error
Frequency	Intercept	4.90066	0.0072
	Slope	-0.0051	1.90161E-4

Statistics

	Frequency
Number of Points	41
Degrees of Freedom	39
Residual Sum of Squares	188.20316
Pearson's r	-0.97391
Adj. R-Square	0.94718

Summary

	Intercept		Slope		Statistics
	Value	Standard Error	Value	Standard Error	Adj. R-Square
Frequency	4.90066	0.0072	-0.0051	1.90161E-4	0.94718

ANOVA

		DF	Sum of Squares	Mean Square	F Value	Prob>F
Frequency	Model	1	3466.03691	3466.03691	718.24212	0
	Error	39	188.20316	4.82572		
	Total	40	3654.24007			

At the 0.05 level, the slope is significantly different from zero.

Fitted Curves Plot

Frequency

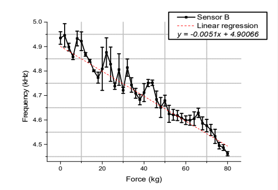

Residual vs. Independent Plot

Frequency

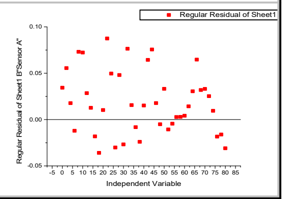

Supplement: S2 Table — (PDF) [file pone.0330422.s004.pdf]
