# Supplementary material for: Flexible PVDF sensors for bruxism bite force measurement: A redefined instrumental approach
Source: PLoS One. 2025 Aug 21;20(8):e0330422. doi: 10.1371/journal.pone.0330422 (PMC12370117; doi:10.1371/journal.pone.0330422)

Linear Fit (30/10/2024 13:45:20)

Parameters

		Value	Standard Error
Capacitance	Intercept	23.81081	0.10838
	Slope	0.03283	0.00233

Statistics

	Capacitance
Number of Points	41
Degrees of Freedom	39
Residual Sum of Squares	4.86978
Pearson's r	0.9141
Adj. R-Square	0.83136

Summary

	Intercept		Slope		Statistics
	Value	Standard Error	Value	Standard Error	Adj. R-Square
Capacitance	23.81081	0.10838	0.03283	0.00233	0.83136

ANOVA

		DF	Sum of Squares	Mean Square	F Value	Prob>F
Capacitance	Model	1	24.74773	24.74773	198.19414	1.11022E-16
	Error	39	4.86978	0.12487		
	Total	40	29.61751			

At the 0.05 level, the slope is significantly different from zero.

Fitted Curves Plot

Capacitance

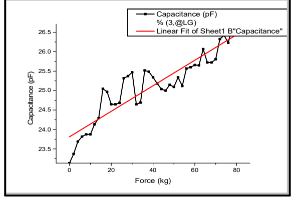

Residual vs. Independent Plot

Capacitance

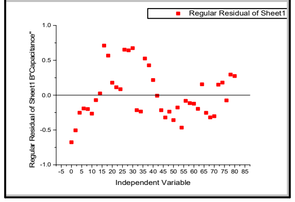

Supplement: S3 Table — (PDF) [file pone.0330422.s005.pdf]
