# Supplementary material for: Flexible PVDF sensors for bruxism bite force measurement: A redefined instrumental approach
Source: PLoS One. 2025 Aug 21;20(8):e0330422. doi: 10.1371/journal.pone.0330422 (PMC12370117; doi:10.1371/journal.pone.0330422)

Linear Fit (30/10/2024 13:59:58)

Parameters

		Value	Standard Error
Capacitance	Intercept	16.19923	0.0368
	Slope	0.01949	7.91893E-4

Statistics

	Capacitance
Number of Points	41
Degrees of Freedom	39
Residual Sum of Squares	0.56153
Pearson's r	0.96927
Adj. R-Square	0.93794

Summary

	Intercept		Slope		Statistics
	Value	Standard Error	Value	Standard Error	Adj. R-Square
Capacitance	16.19923	0.0368	0.01949	7.91893E-4	0.93794

ANOVA

		DF	Sum of Squares	Mean Square	F Value	Prob>F
Capacitance	Model	1	8.71849	8.71849	605.53097	0
	Error	39	0.56153	0.0144		
	Total	40	9.28002			

At the 0.05 level, the slope is significantly different from zero.

Fitted Curves Plot

Capacitance

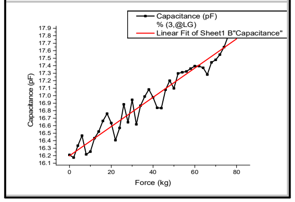

Residual vs. Independent Plot

Capacitance

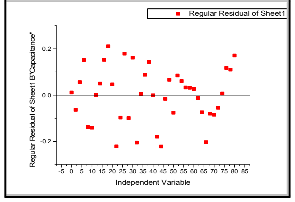

Supplement: S4 Table — (PDF) [file pone.0330422.s006.pdf]
