# Supplementary material for: Flexible PVDF sensors for bruxism bite force measurement: A redefined instrumental approach
Source: PLoS One. 2025 Aug 21;20(8):e0330422. doi: 10.1371/journal.pone.0330422 (PMC12370117; doi:10.1371/journal.pone.0330422)

Linear Fit (30/10/2024 14:17:42)

Parameters

		Value	Standard Error
Frequency	Intercept	2.75935	0.06508
	Slope	-0.00523	0.00672

Statistics

	Frequency
Number of Points	13
Degrees of Freedom	11
Residual Sum of Squares	2215.52582
Pearson's r	-0.22843
Adj. R-Square	-0.03399

Summary

	Intercept		Slope		Statistics
	Value	Standard Error	Value	Standard Error	Adj. R-Square
Frequency	2.75935	0.06508	-0.00523	0.00672	-0.03399

ANOVA

		DF	Sum of Squares	Mean Square	F Value	Prob>F
Frequency	Model	1	121.96663	121.96663	0.60556	0.45288
	Error	11	2215.52582	201.41144		
	Total	12	2337.49246			

At the 0.05 level, the slope is NOT significantly different from zero.

Fitted Curves Plot

Frequency

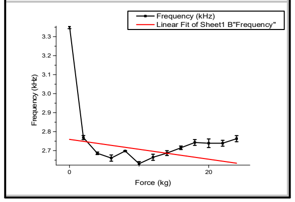

Residual vs. Independent Plot

Frequency

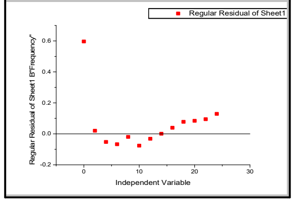

Supplement: S5 Table — (PDF) [file pone.0330422.s007.pdf]
