# Supplementary material for: Flexible PVDF sensors for bruxism bite force measurement: A redefined instrumental approach
Source: PLoS One. 2025 Aug 21;20(8):e0330422. doi: 10.1371/journal.pone.0330422 (PMC12370117; doi:10.1371/journal.pone.0330422)

Linear Fit (30/10/2024 15:15:18)

Parameters

		Value	Standard Error
Frequency	Intercept	3.86559	0.1646
	Slope	-0.01019	0.00863

Statistics

	Frequency
Number of Points	13
Degrees of Freedom	11
Residual Sum of Squares	3136.9398
Pearson's r	-0.33528
Adj. R-Square	0.03173

Summary

	Intercept		Slope		Statistics
	Value	Standard Error	Value	Standard Error	Adj. R-Square
Frequency	3.86559	0.1646	-0.01019	0.00863	0.03173

ANOVA

		DF	Sum of Squares	Mean Square	F Value	Prob>F
Frequency	Model	1	397.3036	397.3036	1.39319	0.26276
	Error	11	3136.9398	285.17635		
	Total	12	3534.2434			

At the 0.05 level, the slope is NOT significantly different from zero.

Fitted Curves Plot

Frequency

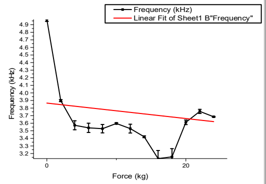

Residual vs. Independent Plot

Frequency

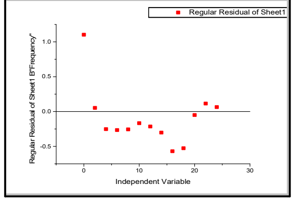

Supplement: S6 Table — (PDF) [file pone.0330422.s008.pdf]
