# Supplementary material for: Flexible PVDF sensors for bruxism bite force measurement: A redefined instrumental approach
Source: PLoS One. 2025 Aug 21;20(8):e0330422. doi: 10.1371/journal.pone.0330422 (PMC12370117; doi:10.1371/journal.pone.0330422)

Linear Fit (30/10/2024 15:43:28)

Parameters

		Value	Standard Error
Capacitance	Intercept	28.2437	0.84851
	Slope	0.07218	0.06

Statistics

	Capacitance
Number of Points	13
Degrees of Freedom	11
Residual Sum of Squares	28.82784
Pearson's r	0.34097
Adj. R-Square	0.03592

Summary

	Intercept		Slope		Statistics
	Value	Standard Error	Value	Standard Error	Adj. R-Square
Capacitance	28.2437	0.84851	0.07218	0.06	0.03592

ANOVA

		DF	Sum of Squares	Mean Square	F Value	Prob>F
Capacitance	Model	1	3.79254	3.79254	1.44714	0.25424
	Error	11	28.82784	2.62071		
	Total	12	32.62039			

At the 0.05 level, the slope is NOT significantly different from zero.

Fitted Curves Plot

Capacitance

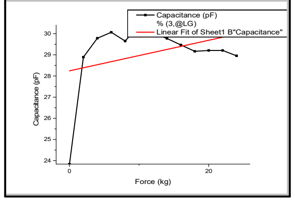

Residual vs. Independent Plot

Capacitance

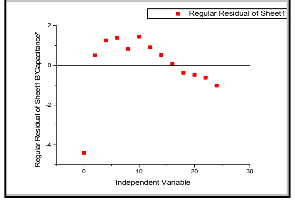

Supplement: S7 Table — (PDF) [file pone.0330422.s009.pdf]
