# Supplementary material for: Flexible PVDF sensors for bruxism bite force measurement: A redefined instrumental approach
Source: PLoS One. 2025 Aug 21;20(8):e0330422. doi: 10.1371/journal.pone.0330422 (PMC12370117; doi:10.1371/journal.pone.0330422)

Linear Fit (30/10/2024 15:59:15)

Parameters

		Value	Standard Error
Capacitance	Intercept	20.5	1.11153
	Slope	0.14188	0.0786

Statistics

	Capacitance
Number of Points	13
Degrees of Freedom	11
Residual Sum of Squares	49.46979
Pearson's r	0.47805
Adj. R-Square	0.1584

Summary

	Intercept		Slope		Statistics
	Value	Standard Error	Value	Standard Error	Adj. R-Square
Capacitance	20.5	1.11153	0.14188	0.0786	0.1584

ANOVA

		DF	Sum of Squares	Mean Square	F Value	Prob>F
Capacitance	Model	1	14.65432	14.65432	3.2585	0.09847
	Error	11	49.46979	4.49725		
	Total	12	64.12411			

At the 0.05 level, the slope is NOT significantly different from zero.

Fitted Curves Plot

Capacitance

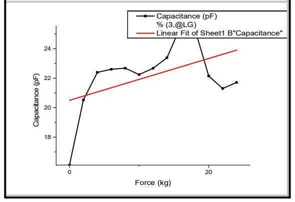

Residual vs. Independent Plot

Capacitance

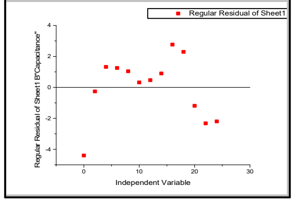

Supplement: S8 Table — (PDF) [file pone.0330422.s010.pdf]
